# Use of copper-cysteamine nanoparticles to simultaneously enable radiotherapy, oxidative therapy and immunotherapy for melanoma treatment

**DOI:** 10.1038/s41392-020-0156-4

**Published:** 2020-05-15

**Authors:** Qi Zhang, Xiangdong Guo, Yingnan Cheng, Lalit Chudal, Nil Kanatha Pandey, Jieyou Zhang, Lun Ma, Qing Xi, Guangze Yang, Ying Chen, Xin Ran, Chengzhi Wang, Jingyi Zhao, Yan Li, Li Liu, Zhi Yao, Wei Chen, Yuping Ran, Rongxin Zhang

**Affiliations:** 1 Department of Immunology and Research Center of Basic Medical Sciences, Key Laboratory of Immune Microenvironment and Diseases of Educational Ministry of China, Tianjin Key Laboratory of Cellular and Molecular Immunology, Tianjin Medical University, 300070 Tianjin, China; 2 Institute of Integrative Medicines for Acute Abdominal Diseases, Tianjin Nankai Hospital, 300100 Tianjin, China; 3 Department of Physics, The University of Texas at Arlington, Arlington, TX 76019-0059 USA; 4 Guangdong Province Key Laboratory for Biotechnology Drug Candidates, Institute of Basic Medical Sciences, School of Life Sciences and Biopharmaceutics, Guangdong Pharmaceutical University, Guangzhou, China; 5 Department of Dermatovenereology, West China Hospital, Sichuan University, Chengdu, Sichuan Province China; 6 Department of Radiology, The University of Texas Southwestern Medical Center, Dallas, TX USA

**Keywords:** Skin cancer, Skin cancer

**Dear Editor**,

Melanoma, squamous cell carcinoma (SCC), and basal cell carcinoma (BCC) are three major types of skin cancer. Among them, melanoma is the most severe form and accounts for ~4% of all newly diagnosed cancers annually in the United States. It is estimated that approximately 9500 people are diagnosed with skin cancer every day, and more than 1 million Americans are living with melanoma. Melanoma treatment is still a major challenge in the clinic. Photodynamic therapy (PDT) is composed of targeted ablation and immune activation, is less invasive than other therapies and has been widely used in the treatment of various cancers. However, the limitation of light penetration is an issue in PDT for deep cancer treatment.^1^ To overcome this limitation and enable PDT for deep cancer treatment, researchers have proposed X-ray-induced PDT^1^ and nanoparticle self-lighting PDT,^2^ and these techniques have become intensively studied topics. Recently, Chen et al.^3^ invented a new sensitizer called copper-cysteamine (Cu–Cy) that can be activated by UV,^4^ X-rays,^5^ microwave,^6^ and ultrasound^7^ to generate reactive oxygen species (ROS) to destroy cancer cells as well as bacteria.^8^ As ROS generation by Cu–Cy nanoparticles (NPs) is not solely activated by regular light, it is more appropriate to call it oxidative therapy (OT) rather than PDT.

Cu–Cy NPs of an average size of 96 nm have been tested for skin cancer treatment.^9^ It was found that these Cu–Cy NP-based X-PDT exhibited a strong antitumor effect towards SCC. However, B16F10 melanoma was resistant to these Cu–Cy NP-based X-PDT, both in vitro and in vivo.^9^

Size is known to be a sensitive factor influencing nanomaterial properties and performance. To further evaluate the effect of Cu–Cy NP-based X-PDT on melanoma, we applied particles with an average size of ~40 nm for the treatment of melanoma, as the 40 nm Cu–Cy NPs have a larger surface area than other NPs, thereby producing more ROS.^10^ In addition, the cell uptake is higher for the 40 nm NPs. As expected, the 40 nm Cu–Cy NPs were very effective in inhibiting melanoma under X-ray stimulation. These observations confirmed that the combination of Cu–Cy and X-rays facilitated cell apoptosis and/or necrosis of B16 cells. More interestingly, this combination promoted the formation of the antitumor immune response. These results suggest that Cu–Cy NPs can simultaneously facilitate radiotherapy, oxidative therapy, and immunotherapy for melanoma treatment, as illustrated in Fig. 1a.

**Fig. 1:**
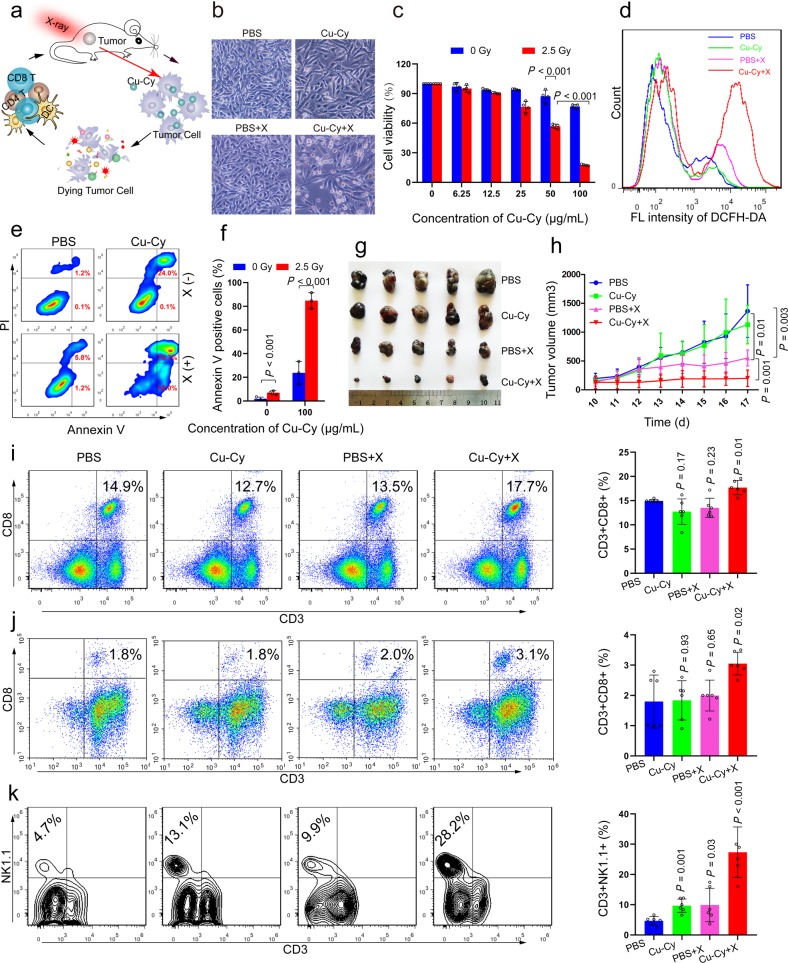
Cu–Cy NP-based X-ray-induced oxidative and antitumor immune responses in melanoma. **a** Schematic illustration of Cu–Cy NPs to simultaneously enable radiotherapy, oxidative therapy, and immunotherapy for melanoma treatment. **b** Morphological images of B16 cells incubated with Cu–Cy NPs (~40 nm) upon X-ray irradiation (0 or 2.5 Gy) revealed significant damage to the cellular morphology in the Cu–Cy NP plus X-ray group compared to the other groups. **c** Cell viability of the B16 cells incubated with different concentrations of Cu–Cy NPs upon 2.5 Gy X-ray irradiation. Data are presented as the mean ± SD. Error bars denote the S.D. **d** Intracellular ROS were significantly increased when the B16 cells were treated with Cu–Cy and X-rays compared to that of other groups. **e**, **f** The highest cell apoptosis and/or necrosis rates were found when the B16 cells were treated with Cu–Cy and X-rays compared to those of the other groups. Data are presented as the mean ± SD. Error bars denote the S.D. **g** Tumor volumes in different groups at the end of treatment. **h** Tumor growth curves in different groups. Data are presented as the mean ± SD. Error bars denote the S.D. (*n* = 6). **i–k** Tumor tissues were removed from the mice to detect changes in the infiltrative immune cells by flow cytometry. **i** The percentage of CD8^+^T cells was significantly enhanced in spleens when the mice were treated with Cu–Cy and X-rays compared to those of the other groups. Data are presented as the mean ± SD. Error bars denote the S.D. *P*-value vs. the PBS group. (*n* = 6). **j**, **k** The percentages of CD8^+^T and NK cells in tumor tissues when the mice were treated with Cu–Cy and X-rays compared to those of the other groups. Data are presented as the mean ± SD. Error bars denote the S.D. *P*-value vs. the PBS group. (*n* = 6)

The distribution of Cu–Cy was assessed by confocal fluorescence microscopy. As shown in Supplementary Fig. S1, the uptake of Cu–Cy NPs in the nucleus after 6 h was substantially increased compared to that after 2 and 4 h of incubation. Next, the cytotoxicity was measured to assess the efficacy of Cu–Cy on B16 cells by the CCK8 viability assay. After incubation with various amounts of Cu–Cy for 24 h, the cells were irradiated with X-rays at 0 or 2.5 Gy. The results showed that the viability of the cells in the control group (Cu–Cy only) had no obvious reduction. In contrast, a dramatic reduction in cell viability was observed in a dose-dependent manner in the 2.5 Gy group (Fig. 1b, c), suggesting that the Cu–Cy NPs had low toxicity towards cells but could easily be activated by X-rays to induce substantial cytotoxicity.

As shown in Fig. 1d and Supplementary Fig. S2a, the Cu–Cy+X-ray group exhibited substantially higher green fluorescence of DCF than the PBS, Cu–Cy, and PBS+X-ray groups, indicating the generation of significant levels of ROS in the Cu–Cy+X-ray group. To further investigate the effect of Cu–Cy-based PDT on B16 cells, we performed a cell apoptosis assay. The apoptosis rate was only 24.1% in the Cu–Cy group (100 µg/mL). In contrast, a significant killing effect was observed in the B16 cells after the combined treatment of Cu–Cy (100 µg/mL) and X-rays with a cell apoptosis rate of ~84.7% (Fig. 1e, f).

To assess the PDT therapeutic efficacy of Cu–Cy in vivo, we subcutaneously injected mice with B16 cells and randomly divided them into four groups. When the tumor volumes reached approximately 300 mm^3^, all mice were intratumorally injected with PBS or Cu–Cy. At 6 h post-injection, the tumor-bearing mice were irradiated by X-rays (5 Gy) in the tumor location. The results demonstrated that the PBS and Cu–Cy groups showed a negligible effect on tumor growth, while the PBS+X-ray and Cu–Cy+X-ray groups showed significant inhibitory efficiency from day 12. Notably, the most significant inhibitory efficiency was found in the Cu–Cy+X-ray group (Fig. 1g, h, and Supplementary Fig. S3c). However, the body weights of the mice did not show obvious changes, and pathological injury to the spleen was not observed in any group (Supplementary Fig. S3b and d). Thus, these results showed that the antitumor activity of the Cu–Cy NPs could be significantly magnified after irradiation with X-rays.

The generation of ROS was the major process in PDT, which could lead to the destruction of tumor cells and further induce the immune response. Considering the obvious antitumor effect of Cu–Cy-based PDT in vivo, we then evaluated whether Cu–Cy-based PDT could trigger an immune response and influence the proportion of immunocytes in the tumor and spleen. Our results revealed that only treatment with Cu–Cy+X-rays triggered the enhancement of CD4^+^T and CD8^+^T cells in the spleen (Fig. 1i and Supplementary Fig. S4a), while no noticeable change was observed in DCs, macrophages, neutrophils, NK cells, and γδT cells in the spleen or MDSCs with slight changes (Supplementary Fig. S6).

The recruitment of immune cells into the tumor microenvironment (TME) is an important event associated with antitumor immune responses. Thus, we investigated whether the antitumor effect of Cu–Cy-based PDT was facilitated by an increase in the infiltration of immune cells, including DCs, M1 macrophages, CD4^+^T cells, and NK cells, in tumors. Flow cytometric analysis showed that DCs, CD8^+^T cells, and NK cells displayed the highest proportion in Cu–Cy+X-ray-treated tumor tissues (Fig. 1j, k and Supplementary Fig. S4b–d). Mature DCs play a key role in initiating an effective adaptive immune response by presenting antigens to T lymphocytes. Previous studies have shown that mature DCs could activate NK cells, while activated NK cells in turn facilitated mature DCs. It has also been found that activated NK cells can directly kill tumor cells and may also control the levels of intratumoral stimulatory dendritic cells (SDCs) in the tumor microenvironment (TME), further increasing the capacity of antigen-presenting DCs and inducing the responses of T and B cells against B16 melanoma. In addition, compared with the PBS-, Cu–Cy-, and PBS+X-ray-treated groups, PDT strongly attenuated the number of M2 macrophages in the Cu–Cy+X-ray group, while the proportion of M1 and MDSC macrophages was not obviously changed (Supplementary Fig. S7). Collectively, our results indicated that Cu–Cy-mediated PDT could induce potent antitumor immune responses via DC maturation; subsequent activation of CD4^+^T cells, CD8^+^T cells, and NK cells; and inhibition of M2 macrophages in the TME, which inhibited tumor growth by killing or suppressing tumor cells.

In summary, upon X-ray activation, Cu–Cy NPs can produce substantial levels of ROS, leading to the direct destruction of melanoma. In addition to ROS generation, Cu–Cy NPs plus X-rays can effectively induce an antitumor immune response. Overall, Cu–Cy NPs can simultaneously enable radiotherapy, oxidative therapy, and immunotherapy for cancer treatment and help to overcome the limitations of traditional cancer treatment modalities.

## Supplementary information

Supplementary materials in PDF
