# Supplementary material for: Use of copper-cysteamine nanoparticles to simultaneously enable radiotherapy, oxidative therapy and immunotherapy for melanoma treatment
Source: Signal Transduct Target Ther. 2020 May 15;5:58. doi: 10.1038/s41392-020-0156-4 (PMC7225170; doi:10.1038/s41392-020-0156-4)
Supplement: Supplementary file 1 — Supplementary materials in PDF [file 41392_2020_156_MOESM1_ESM.pdf]

Supporting Information

Use of copper-cysteamine nanoparticles to simultaneously enable radiotherapy, oxidative therapy and immunotherapy for melanoma treatment

Qi Zhang[2,1,#], Xiangdong Guo[1,#], Yingnan Cheng[1,#], Lalit Chudal[3], Nil Kanatha Pandey[3], Jieyou Zhang[1], Lun Ma[3], Qing Xi[1], Guangze Yang[1], Ying Chen[3], Xin Ra5n, Chengzhi Wang[1], Jingyi Zhao[4], Yan Li[1], Li Liu[6], Zhi Yao[1], Wei Chen[3,*] Yuping Ran[5*], and Rongxin Zhang[4,1,*]

[1]Department of Immunology and Research Center of Basic Medical Sciences, Key Laboratory of Immune Microenvironment and Diseases of Educational Ministry of China, Tianjin Key Laboratory of Cellular and Molecular Immunology, Tianjin Medical University, Tianjin 300070, China

[2]Institute of Integrative Medicines for Acute Abdominal Diseases, Tianjin Nankai Hospital, Tianjin 300100, China

[3]Department of Physics, The University of Texas at Arlington, Arlington, Texas, 76019-0059, USA

[4]Guangdong Province Key Laboratory for Biotechnology Drug Candidates, Institute of Basic Medical Sciences, School of Life Sciences and Biopharmaceutics, Guangdong Pharmaceutical University, Guangzhou, China

[5]Department of Dermatovenereology, West China Hospital, Sichuan University, Chengdu, Sichuan Province, China

[6]Department of Radiology, The University of Texas Southwestern Medical Center, Dallas, Texas, USA.

[#] These authors contributed equally to this work.

*Corresponding authors: Wei Chen (weichen@uta.edu); Rongxin Zhang (rxzhang@tmu.edu.cn); Yuping Ran (ranyuping@vip.sina.com)

Supporting Information

Materials and Methods

Animals

Female C57BL/6 mice (8 weeks old) were obtained from the Academy of Military Medical Science (Beijing, China). All animals were housed and fed in a specific pathogen-free animal facility at the Experimental Animal Center of Tianjin Medical University (Tianjin, China). The experiments were carried out following the guidelines for animal care approved by the Animal Ethics Committee of Tianjin Medical University (Tianjin, China).

Cu-Cy nanoparticles and cell culture

Cu-Cy nanoparticles were synthesized in Wei Chen's lab at The University of Texas at Arlington, USA.[1] B16 mouse melanoma cell line was obtained from the Cell Resource Center, Peking Union Medical College. All cells were cultured in Dulbecco modified Eagle medium (DMEM) (Hyclone, USA) supplemented with 10 % FBS (HyClone, GE) penicillin (100 U/mL), and streptomycin (100 μg/mL) at 37 ℃ with 5 % CO_2.

X-ray irradiation

The X-ray system was obtained from Faxitron X-Ray Corp (IL, USA). X-ray irradiation (160 kV and 25 mA) was performed at a dose rate of 2.5 Gy *in vitro* or 5 Gy *in vivo*.

Cellular uptake of Cu-Cy

B16 cells (2×10^5 cells/well) were seeded into 12-well plates and incubated at 37 ℃ for 12 h. Cells were incubated with Cu-Cy at the concentration of 100 μg/mL for the indicated times (2, 4, and 6 h). Subsequently, the medium was removed and the cells were washed with PBS three times. Cells were then fixed with 4 % paraformaldehyde for 10 min at 4 °C and stained with DAPI for 1 h in dark condition. The images were taken on an Olympus FluoView FV1000 microscope.

X-ray treatment

When the density of cells reached about 70 %, they were pretreated using various amounts of Cu-Cy (0, 6.25, 12.5, 25, 50, and 100 μg/mL) in the complete culture medium. After 6 h of incubation, the

medium was replaced with fresh culture medium and cells were irradiated with 0 or 2.5 Gy X-ray and incubated for another 24 h.

CCK-8 cell viability assay

The cell viability was evaluated by the CCK-8 colorimetric assay (Dojindo, Minato-ku, Tokyo, Japan). B16 cells were plated in 24-well plates at 2×10^4 cells/well for 12 h at 37 °C. The old medium was removed and fresh media containing different concentrations of Cu-Cy (0, 6.25, 12.5, 25, 50, and 100 μg/mL) were added into the wells and incubated for 24 h. Afterward, the cells were washed with PBS three times to remove the free nanoparticles. Then, the cells were treated with X-ray (0 or 2.5 Gy) and incubated for another 24 h. Finally, the medium was replaced with 100 μL mixed medium (DMEM: CCK8 = 9:1) and incubated for 4 h. The absorbance was measured at 490 nm via scanning multiwell spectrophotometer.

ROS detection *in vitro*

The intracellular ROS was detected by DCFH-DA using an Olympus FluoView FV1000 microscope and flow cytometry following the manufacturer's protocol. For flow cytometry analysis of ROS, B16 cells were seeded into 12-well plates at 2×10^4 cells/well for 12 h and then incubated with PBS and Cu-Cy (100 μg/mL) for 6 h. Then, cells were irradiated with X-ray (2.5 Gy) and co-cultured with DCFH-DA (10 μM) diluted with serum-free medium (1:1000) for 30 min. The culture medium was then removed and washed for three times with serum-free medium. Afterward, cells were collected and the fluorescence signals of ROS intensity were analyzed by flow cytometry or Olympus FluoView FV1000 microscope.

Apoptosis assay

The cellular apoptosis was assessed by the Annexin V-FITC/PI Apoptosis Detection Kit (BD Pharmingen, San Jose, CA, USA). 2×10^5 cells/well were cultured in 12-well plates for 12 h. After the Cu-Cy (0 and 100 μg/mL) and X-ray treatments, the cells were harvested and washed with PBS for two times, then resuspended with 1 mL of 1x binding buffer, stained with 5 μL Annexin V-FITC for 10 min at room temperature in the dark, and then stained with 5 μL of PI for 5 min in the dark. Finally,

these cells were analyzed by a FACSCalibur instrument (BD Biosciences). The results were analyzed with FlowJo software (Tree Star, Ashland, OR).

Western blot analysis

After PDT, B16 cells were lysed in RIPA buffer containing 1 % phosphatase inhibitor cocktail and 1 mM PMSF for 30 min. 20 μg protein was subjected to SDS-PAGE and transferred onto PVDF membranes for western blot analysis using rabbit p-STAT3(705), STAT3, Bcl2, Bax, Cytochrome C, mouse, anti-GAPDH antibodies (Cell Signaling Technology). All of the antibodies were diluted with 1:1000. The ECL Western Blotting Detection System (Millipore Corporation, Billerica, MA, USA) was used to detect immunoreactive bands.

Therapeutic efficacy analysis

B16 cells (2×10^5) were injected subcutaneously into the mice (6-8 weeks old, n = 6). The growth of the tumor was measured every 2 days. When tumor volume reached 300 mm^3, the mice were randomly divided into four groups: PBS, Cu-Cy, PBS+X-ray, and Cu-Cy+X-ray groups. Afterward, mice were anesthetized and injected 50 μL PBS or 50 μL Cu-Cy (50 μg/mL) intratumorally. In X-ray groups, at 6 h post-injection, the mice were irradiated with X-ray (5 Gy) on the tumor location for a total of three times within a week. The size of the tumor and weight of mice were measured daily. The formula: volume = $1/2\times LW^2$ (L and W represent the length and width of the tumor, respectively) was used to calculate the volume of the tumor. After 10 days from the first treatment, the mice were sacrificed. The weight of the tumors was measured. The main organs (heart, liver, spleen, lung, and kidney) and tumor were collected and used for hematoxylin and eosin (H & E)-stained, while the spleen and tumor were used for flow cytometry analysis.

Flow cytometry analysis

All tumor and spleen were harvested, the tumor was mechanically cut into small pieces and digested with collagenase and hyaluronidase at 37 °C. After 1 h, the tumor tissue suspension was ground and filtered with 40 μM filters in order to obtain one single-cell suspension. For analysis of the different types of immune cells, DCs were stained with anti-mouse CD11c-APC and anti-mouse MHC II-PE

antibodies; NK cells were stained with anti-mouse CD3-FITC, anti-mouse NK1.1-APC and anti-mouse CD44-PE antibodies; M1 macrophages were stained with anti-mouse CD11b-FITC, anti-mouse F4/80-APC and anti-mouse MHC II-PE antibodies; M2 macrophages were stained with anti-mouse CD11b-FITC, anti-mouse F4/80-APC and anti-mouse CD206-FITC antibodies; $CD4^{+}$T cells were stained with anti-mouse CD3-FITC, anti-mouse CD4-APC and anti-mouse CD44-PE antibodies; $CD8^{+}$T cells were stained with anti-mouse CD3-FITC, anti-mouse CD8-APC and anti-mouse CD44-PE antibodies; Neutrophils were stained with anti-mouse CD11b-FITC, anti-mouse F4/80-APC and anti-mouse Ly6c-PE antibodies; Myeloid-derived suppressor cells (MDSCs) were stained with anti-mouse CD11b-FITC, anti-mouse Ly6G-PECY7 antibodies and anti-mouse Ly6c-PE antibodies; γδT cells were stained with anti-mouse γδT-APC and anti-mouse CD3-FITC antibodies (all antibodies were purchased from eBiosciences, USA). The percent of immune cell populations above were measured with the flow cytometry.

Statistical analysis

All tests were carried out with GraphPad Prism 6 software (GraphPad Software, San Diego, USA). The data were presented as mean ± SD. The statistical analyses were performed by unpaired two-tailed Student's t-tests. $P < 0.05$ was considered statistically significant.

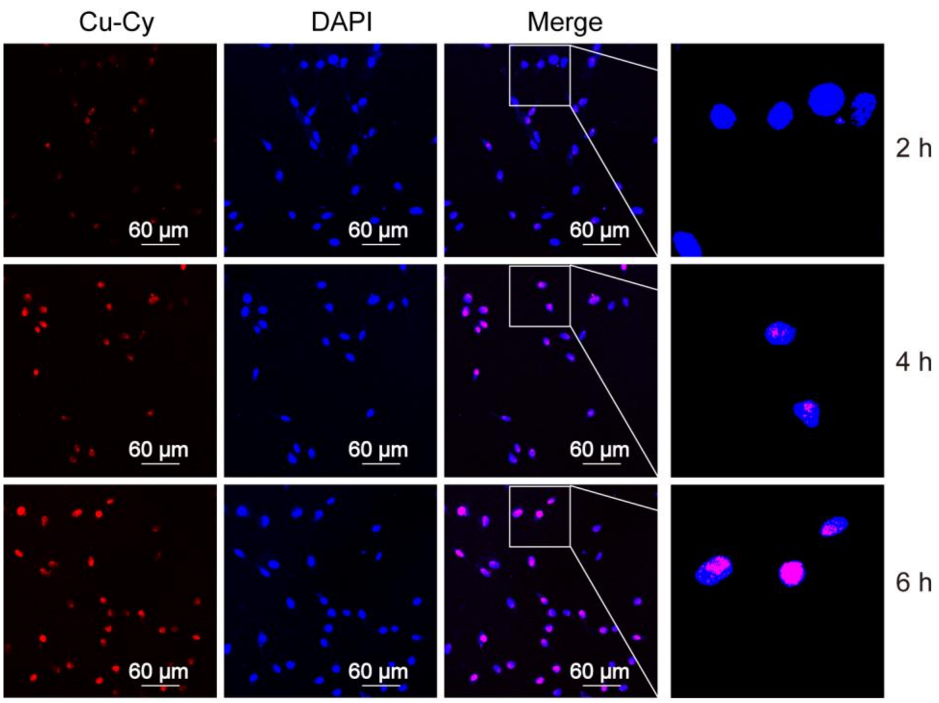

Fig. S1 Cellular uptake of Cu-Cy NPs in B16 cells observed by an Olympus FluoView FV1000 microscope after different incubation times. The enlarged view of the selected area (rightmost column) shows the nuclear uptake of Cu-Cy NPs.

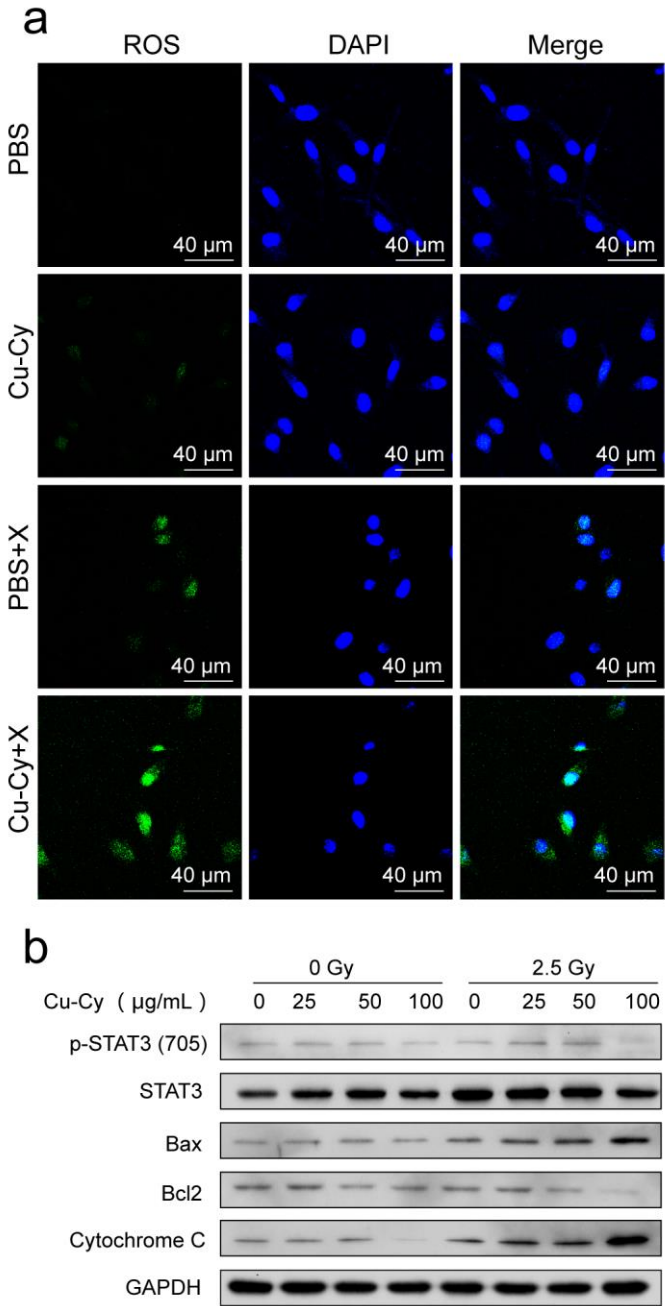

Fig. S2 Cu-Cy-based X-ray induced cell apoptosis and/or necrosis *in vitro*. B16 cells were co-cultured with Cu-Cy and irradiated by 0 or 2.5 Gy X-ray. **a:** Intracellular ROS was significantly increased when B16 cells were treated with Cu-Cy and X-ray as compared to other groups. **b:** Western blot shows that the change of p-STAT3 (705), Bcl2, Bax, and cytochrome C upon Cu-Cy/X-ray treatment.

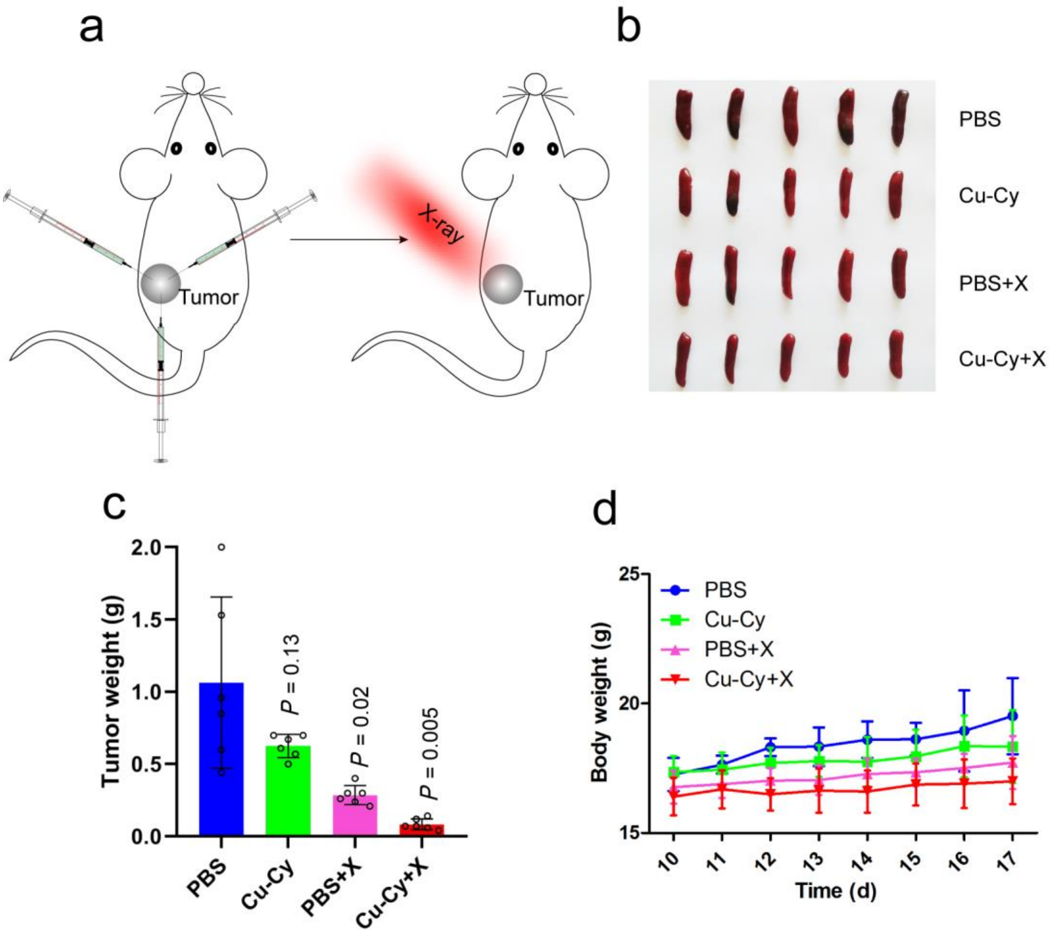

Fig. S3 Therapeutic efficacy of Cu-Cy nanoparticles *in vivo*. a: Schematic illustration of the PDT treatment. **b:** Photographs of spleen from different groups at the end of treatment. **c-d:** Tumor weights and mice body weights in different groups at the end of treatment, respectively. Differences in body weight between mice may be due to differences of tumor weight between different groups. Data are presented as mean ± SD. Error bars donate S.D. *P* value vs. PBS group. (n=6).

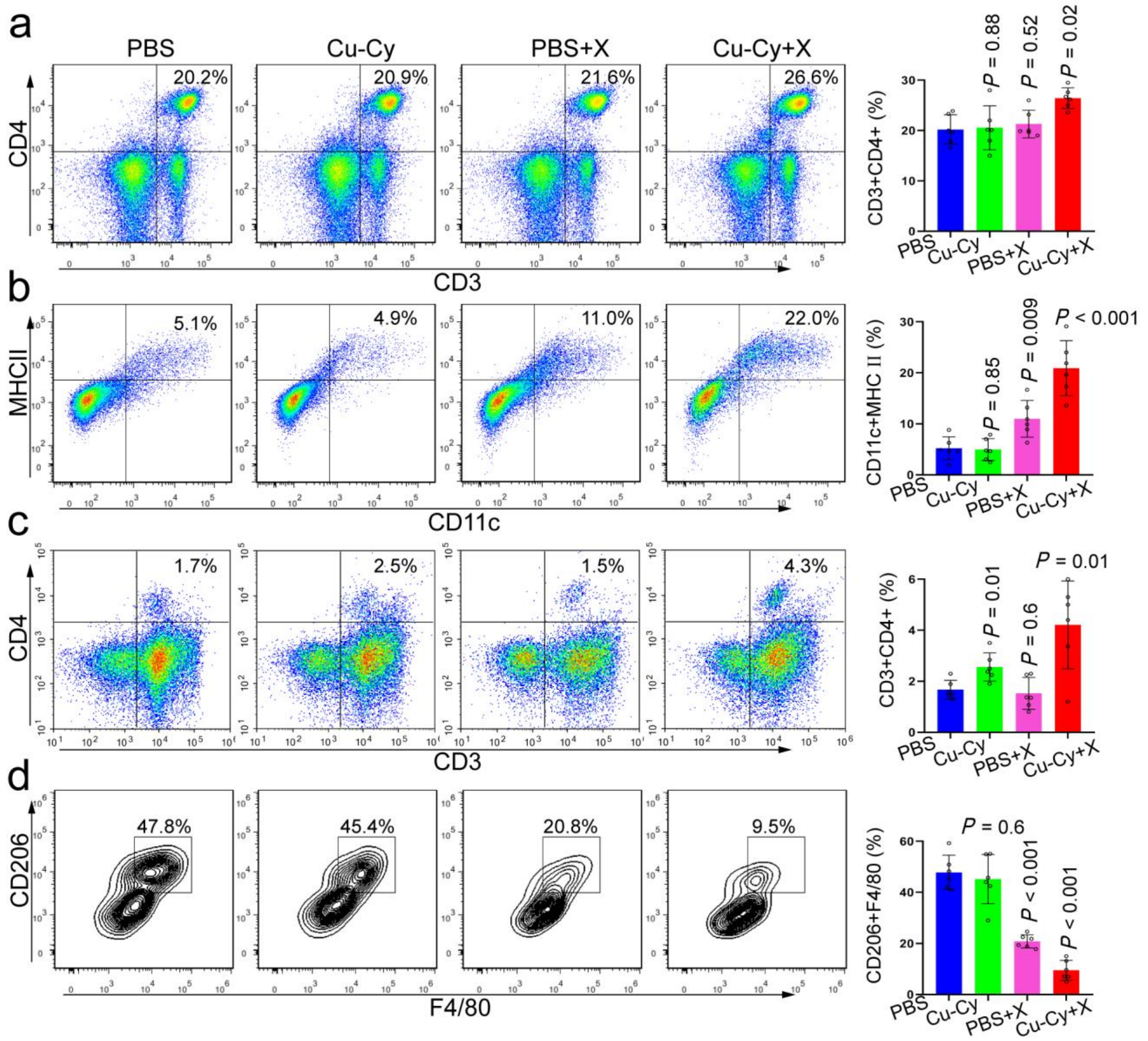

Fig. S4 Cu-Cy-based PDT induced antitumor immune response. Tumor tissues were removed from mice to detect changes in infiltrative immune cells by flow cytometry. **a:** The percentage of CD4$^+$T cells was significantly increased in spleens when mice were treated with Cu-Cy and X-ray compared to other groups. Data are presented as mean ± SD. Error bars donate S.D. *P* value vs. PBS group. (n=6). **b-d:** The percentage of DCs, CD4$^+$ T, and M2 cells in tumor tissues when mice were treated with Cu-Cy and X-ray compared to other groups. Data are presented as mean ± SD. Error bars donate S.D. *P* value vs. PBS group. (n=6).

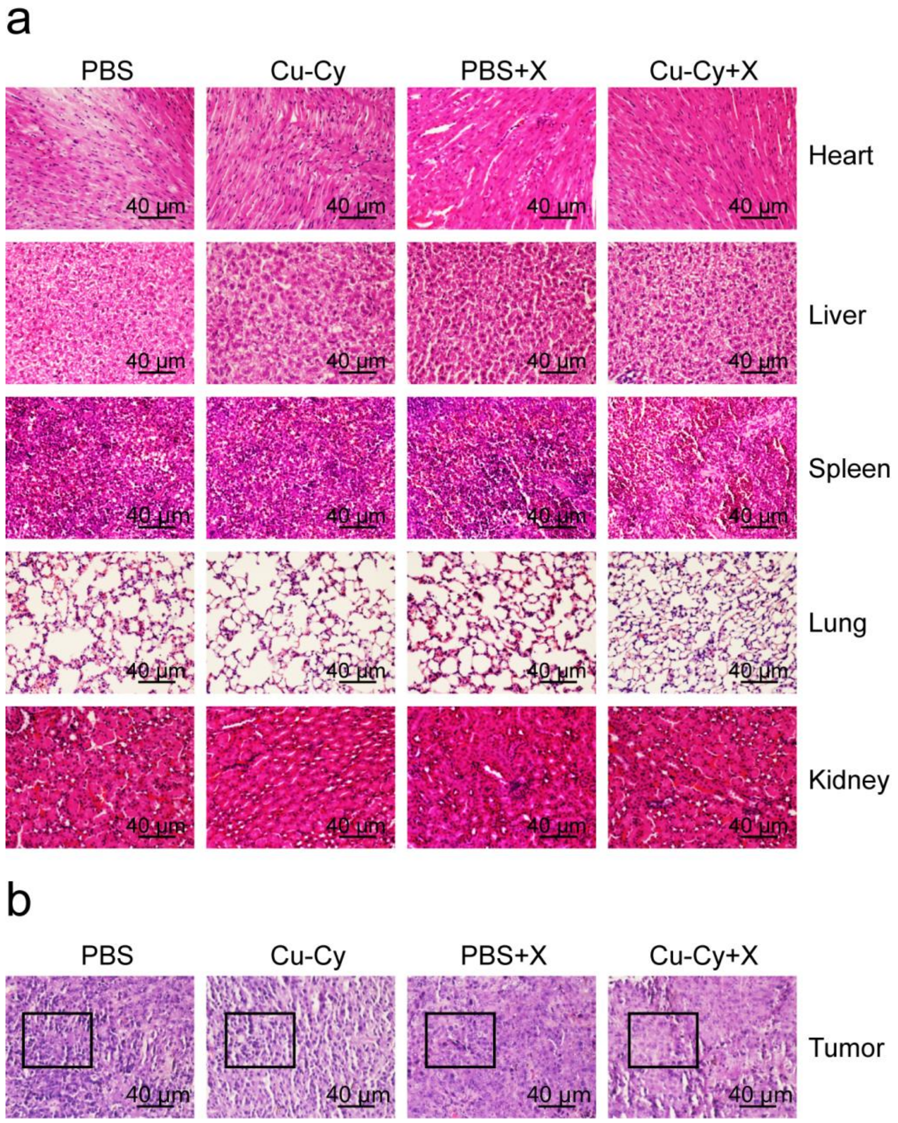

Fig. S5. Biosafety analysis of Cu-Cy in mice body. Mice were sacrificed and major organs and tumor tissues were collected for evaluating biosafety analysis of Cu-Cy in mice body. **a:** H&E staining of heart, liver, spleen, lung, and kidney of tumor-bearing mice treated with PBS, Cu-Cy, PBS+X-ray, and Cu-Cy+X-ray. **b:** H&E staining of tumor tissue of tumor-bearing mice treated with PBS, Cu-Cy, PBS+X-ray, and Cu-Cy+X-ray.

Supporting Information

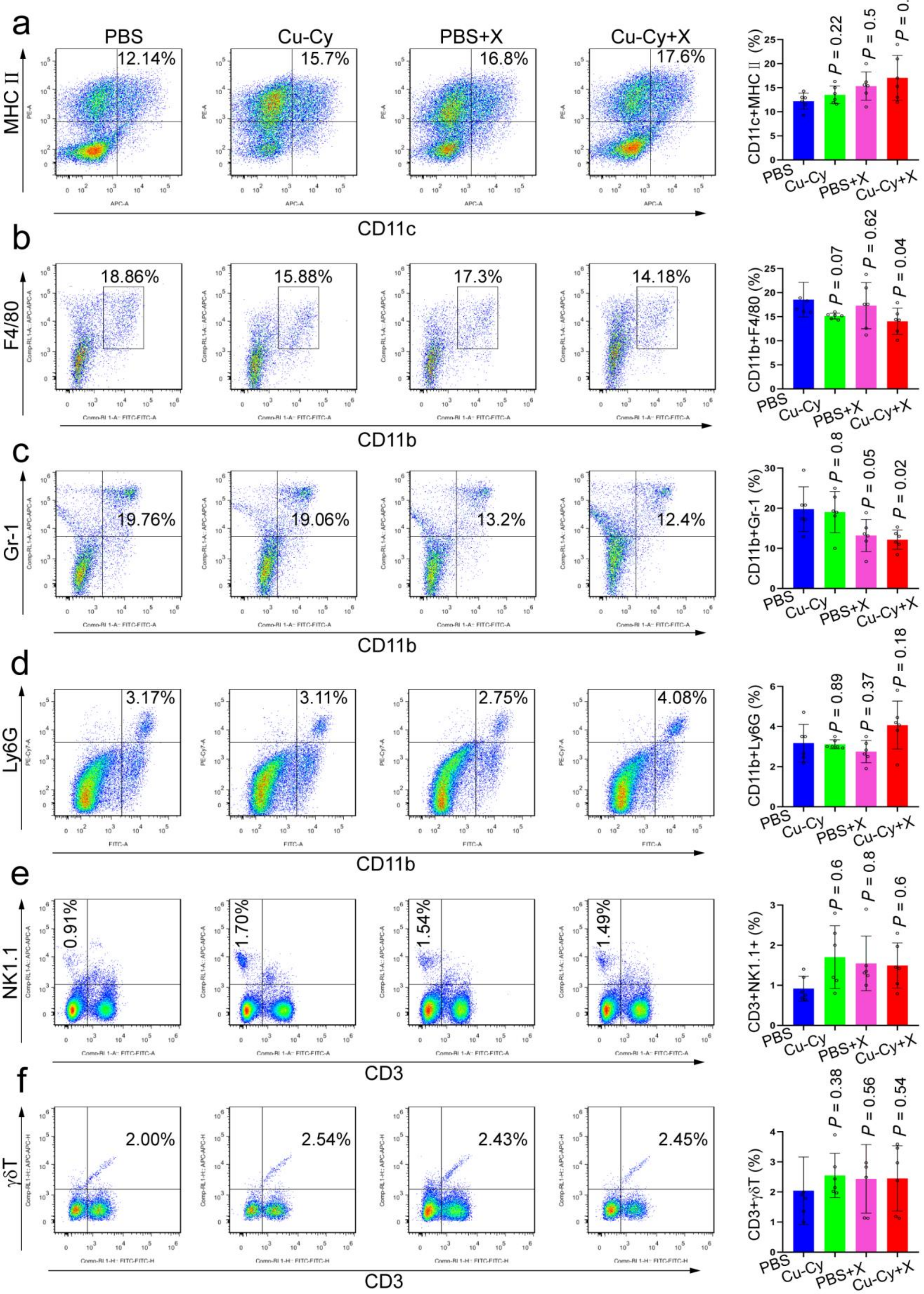

Fig. S6 Cu-Cy-based PDT induced antitumor immune response in the spleen of the mouse. Spleens were removed from mice to detect changes of infiltrative immune cells by flow cytometry. **a-f**: The percentage of DC, macrophages, MDSC, neutrophils, NK, and γδT cells in the spleen of the mouse, respectively. Data are presented as mean ± SD. Error bars donate S.D. *P* value vs. PBS group. (n=6).

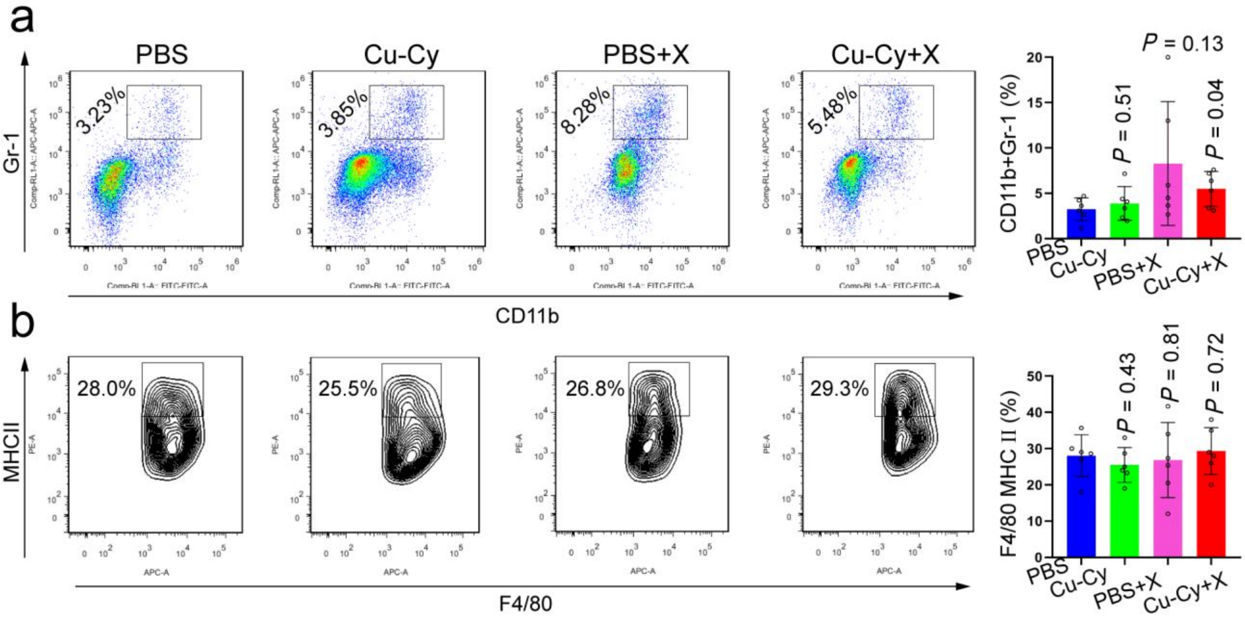

Fig. S7 Cu-Cy-based PDT induced antitumor immune response in the tumor of the mouse. Tumor tissues and spleen were removed from mice to detect changes of infiltrative immune cells by flow cytometry. **a-b**: The percentage of MDSCs and M1 macrophages in the tumor of the mouse. Data are presented as mean ± SD. Error bars donate S.D. *P* value vs. PBS group. (n=6).

Reference

1. Pandey, N.K., *et al.* A facile method for the synthesis of copper-cysteamine nanoparticles and study of ROS production for cancer treatment. *Journal of Materials Chemistry B* **7**, 6630-6642 (2019).
